# The Role of Immunoglobulin Superfamily Cell Adhesion Molecules in Cancer Metastasis

**DOI:** 10.1155/2012/340296

**Published:** 2012-01-09

**Authors:** Chee Wai Wong, Danielle E. Dye, Deirdre R. Coombe

**Affiliations:** Molecular Immunology Group, School of Biomedical Sciences and Curtin Health Innovation Research Institute, Curtin University Level 3 MRF Building, Rear 50 Murray Street, Perth, WA 6000, Australia

## Abstract

Metastasis is a major clinical problem and results in a poor prognosis for most cancers. The metastatic pathway describes the process by which cancer cells give rise to a metastatic lesion in a new tissue or organ. It consists of interconnecting steps all of which must be successfully completed to result in a metastasis. Cell-cell adhesion is a key aspect of many of these steps. Adhesion molecules belonging to the immunoglobulin superfamily (Ig-SF) commonly play a central role in cell-cell adhesion, and a number of these molecules have been associated with cancer progression and a metastatic phenotype. Surprisingly, the contribution of Ig-SF members to metastasis has not received the attention afforded other cell adhesion molecules (CAMs) such as the integrins. Here we examine the steps in the metastatic pathway focusing on how the Ig-SF members, melanoma cell adhesion molecule (MCAM), L1CAM, neural CAM (NCAM), leukocyte CAM (ALCAM), intercellular CAM-1 (ICAM-1) and platelet endothelial CAM-1 (PECAM-1) could play a role. Although much remains to be understood, this review aims to raise the profile of Ig-SF members in metastasis formation and prompt further research that could lead to useful clinical outcomes.

## 1. Introduction

Cell proliferation, migration, and differentiation are critically important during the development of all organisms, and it is the overall coordination of these activities that leads to the formation of complex structures such as tissues and organs. These cellular processes are modulated by the interaction of cells with each other and with their microenvironment. Cell adhesion molecules (CAMs) facilitate these interactions and are essential during development and for maintaining the integrity of tissue architecture in adults [1, 2]. CAMs include cadherins, integrins, selectins, and the immunoglobulin superfamily (IgSF). In normal tissue, CAM expression is tightly regulated. However, aberrant expression of CAMs disrupts normal cell-cell and cell-matrix interactions, freeing cells from normal check points and constraints, and facilitating tumour formation and metastasis [3]. Although much has been written about the role of integrins and cadherins in cancer metastasis, the IgSF has received less attention. Here we explore the roles of some IgSF members in each step of the metastatic cascade.

## 2. Immunoglobulin Superfamily

With over 765 members, the IgSF is one of the largest and most diverse families of proteins in the body. Members of the IgSF include major histocompatibility complex class I and II molecules, proteins of the T cell receptor complex, virus receptors, and cell surface glycoproteins [4]. The definitive characteristic of the IgSF members is the presence of one or more immunoglobulin- (Ig-) like domains, which have a characteristic sandwich structure composed of two opposing antiparallel *β*-pleated sheets, stabilized by a disulphide bridge [5]. Most of the IgSF members are type I transmembrane proteins, which typically consist of an extracellular domain (which contains one or more Ig-like domains), a single transmembrane domain, and a cytoplasmic tail [6]. IgSF members mediate calcium-independent adhesion through their N-terminal Ig-like domains, which commonly bind other Ig-like domains of the same structure on an opposing cell surface (homophilic adhesion) but may also interact with integrins and carbohydrates (heterophilic adhesion) [7]. The C-terminal intracellular domains of IgSF members often interact with cytoskeletal or adaptor proteins. In this way, the extracellular interactions of IgSF CAMs can lead to signaling within the cell, enabling these proteins to function in a wide range of normal biological processes, as well as pathological events such as tumourigenesis.

## 3. The IgSF and Metastasis

A number of IgSF members have been identified as biomarkers for cancer progression. For example, MCAM (also called CD146, Mel-Cam, Muc18, and S-Endo1) has been implicated in the progression of melanoma, as well as in breast and prostate cancer [8–10]. Similarly, IgSF members such as L1CAM (CD171), NCAM (CD56), PECAM-1 (CD31), ALCAM (CD166), and ICAM-1 (CD54) have been associated with metastatic progression in a range of cancers including melanoma, glioma, breast, ovarian, endometrial, prostate, and colon cancer [11–15]. In this paper, we will focus on the roles of these six IgSF members in the metastatic cascade (Table 1).

Metastasis is the endpoint of an evolutionary process in which cells acquire the ability to overcome intrinsic (genomic) and extrinsic (microenvironmental) constraints imposed upon them and hence, are able to escape their preprogrammed behavior [16, 17]. During metastatic spread, tumour cells disseminate to sites distant from the primary tumour, using cell migration mechanisms that are similar, if not identical, to normal physiological processes [18]. The metastatic process consists of five sequential steps: (1) tumor cell proliferation and angiogenesis; (2) local cell invasion; (3) intravasation and dissemination; (4) extravasation; (5) metastatic colonization and proliferation [19]. Tumour cells may also have to withstand immunological attack during any of these stages. IgSF members have been implicated in most, if not all, of these processes.

## 4. Cell Proliferation in the Primary Tumour

### 4.1. Apoptotic Evasion

The first step in metastasis is the transformation of cells from a normal to a cancerous phenotype. This is when cells acquire characteristics that help them to withstand factors that may limit their metastatic spread. These factors include genotypic stress, tissue hypoxia, nutrient depletion, the accumulation of toxic metabolites, haemodynamic shearing, and loss of adhesion [20, 21]. Most cells encountering these factors will undergo apoptosis (preprogrammed cell death) [21, 22]. However, genome expression analysis of metastatic tumours using cDNA microarrays has revealed a strong correlation between tumour progression and the loss of expression of proapoptotic genes, with a concomitant gain in expression of antiapoptotic genes [22]. Thus, the acquisition of apoptotic resistance in cells under stress is the first requirement in tumour progression toward metastasis.

Classically, genotypic stress due to genomic instability through DNA mutation, chromosomal rearrangement, and epigenetic alteration will trigger apoptosis through the tumour suppressor p53 (TS P53) pathway. In many tumour cells, the expression of TS P53 is lost, enabling them to avoid apoptotic death. However, this accounts for only 40% of cells that undergo malignant transformation [23]. Recent reports have indicated that aberrant expression of CAMs such as the IgSF members provides antiapoptotic signals that may account for the other 60% of malignant transformation. For example, Campodónico et al. [24] reported that the functional blockade of NCAM led to susceptibility to apoptosis in murine lung tumour cells and suggested that NCAM expression may be linked to apoptotic resistance in these cells. This resistance seems to be due to activation of the transcription factor, nuclear factor kappa B (NF-*κ*B), whose downstream targets are antiapoptotic genes such as B-cell lymphoma/leukemia-x long (Bcl-x1), X-linked inhibitor of apoptosis protein (XIAP), and cellular inhibitor of apoptosis protein (C-IAP) [12, 25, 26]. MCAM expression by melanoma cells has also been shown to activate NF-*κ*B via the upstream p38 mitogen-activated protein kinase (MAPK) [27]. Inhibition of MCAM using a blocking monoclonal antibody led to downregulation of p38 MAPK phosphorylation, the suppression of NF-*κ*B activation, and a decrease in tumour growth, possibly due to cell death through apoptosis [28]. ALCAM, another member of the IgSF, may also induce apoptotic resistance in tumour cells. For example, siRNA-mediated silencing of ALCAM on the MCF7 breast cancer cell line led to a decrease in the expression of the antiapoptotic protein B-cell-lymphoma- (BCL-) 2 and increased levels of markers of apoptosis [29].

### 4.2. Angiogenesis

After acquiring apoptotic resistance, tumour progression is dependent on the initiation of angiogenesis (the formation of new blood vessels from preexisting vasculature). This process is tightly regulated and involves endothelial cell proliferation, differentiation, and migration, in addition to the degradation of the extracellular matrix [16, 55]. These newly formed blood vessels supply nutrients and oxygen essential for tumour growth. The initiation of angiogenesis is triggered by an imbalance between multiple pro- and antiangiogenic molecules and is known as the “angiogenic switch” [56]. Some of the best-characterized proangiogenic molecules are vascular-endothelial-growth-factor- (VEGF-) A and hypoxia-inducible factor-1 alpha (HIF-1*α*). One of the major characteristics of solid tumours is tissue hypoxia, as the existing blood supply is not sufficient to supply the growing cell mass. Reduced cellular oxygen levels lead to decreased degradation and an accumulation of HIF-1*α* protein in the nucleus of tumour and stromal cells, which initiates transcription of *VEGF* and increases production of VEGF protein [57, 58]. The VEGF secreted by the tumour cells and stroma then stimulates the expression and modulates the function of IgSF members such as ICAM-1, VCAM-1, and PECAM-1 [40, 41, 59] in endothelial cells. For example, ICAM-1-mediated adhesion of leukocytes to endothelia is a key event in early angiogenesis and is also important in the development of endothelial cell polarity, thus mediating endothelial cell migration [33, 34]. VCAM-1 is believed to perform a similar role to that of ICAM-1 [32] while PECAM-1 regulates both endothelial adhesion and migration by modulating endothelial cell-cell and cell-matrix interactions [30, 31].

## 5. Local Invasion

### 5.1. Cell-Cell Interactions

Once tumour growth has reached a critical mass, the metastatic spread of tumour cells is dependent on their dissociation from the primary tumour and migration towards the systemic circulation. Primary tumours with invasive properties usually display reduced intercellular adhesion, which allows cells to break away from the parental cell mass. In tumours arising from epithelial cells, that is, carcinoma, E-cadherin is the major protein involved in cell-cell adhesion. Thus, the loss of E-cadherin expression enables cancer cells to dissociate from the primary tumour and migrate through the extracellular matrix [60, 61]. However, the detachment of cells from a primary tumour is not as simple as the loss of E-cadherin expression. Although some cells migrate as individuals, it has become increasingly clear that cells metastasizing from some solid tumours (e.g., breast and prostate cancer, melanoma, and rhabdomyosarcoma) often migrate together in tightly or loosely associated groups [62]. This suggests that cancer cells retain some cell-cell adhesion, even as they break away from the primary tumour. Other proteins that mediate cell-cell binding include IgSF members such as NCAM, MCAM, ALCAM, and L1CAM. These noncadherin systems are upregulated in cells following the loss of E-cadherin expression and are associated with an active, mobile state that retains enough cell-cell junctions to allow a group of cells to move as a unit [63]. For example, Johnson et al. [8] found an increase in homophilic cell-cell adhesion in melanoma cells transfected with MCAM compared to their MCAM-negative counterparts. Similar reports have indicated that upregulation of ALCAM and L1CAM mediates homophilic cell-cell cohesion in invading melanoma [37] and colorectal carcinoma [14, 38], respectively.

The benefits of collective cell migration include the production of relatively high local concentrations of growth factors, the protection of cells in the centre of a group from immunological attack, and the survival advantage of a mixed population that contains cells able to survive a range of different environmental challenges [18]. In addition, the mechanotransducing force of a migrating cell group exceeds that of a single migrating cell and results in enhanced cell motility.

### 5.2. Directional Cell Migration

Whether or not cells dissociated from the primary tumour migrate individually or collectively, to metastasize they must acquire the ability to migrate towards vascular or lymph vessels [64]. This migration is due, at least in part, to the interaction between chemokine receptors on cancer cells and chemokine gradients in the surrounding tissue. Although malignant cells from different types of cancers express different chemokine receptor profiles, the chemokine receptor most commonly expressed is CXC chemokine receptor 4 (CXCR4), which binds to the CXC chemokine ligand 12 (CXCL12), also known as stromal cell-derived factor-1 alpha (SDF-1*α*) [65]. CXCR4 expression is low or absent in many normal tissues but is expressed by at least 23 different types of tumour cells including cancers of epithelial, mesenchymal, and haemopoietic origin [66, 67]. Its ligand, CXCL12, is found in some primary tumour sites and sites of cancer metastasis and is also constitutively expressed by normal organs such as the bone marrow [65]. *In vitro* experiments have shown that the directional migration of a range of cancer cells (e.g., ovarian, pancreatic, rhabdomyosarcoma and melanoma) is stimulated by the interaction between CXCR4 and CXCL12 [65, 66, 68]. Furthermore, downregulation of CXCR4 through RNA interference or functional blockade using monoclonal antibodies showed a decrease in the invasiveness of breast cancer [69] and melanoma [70].

CXCR4 expression can be upregulated in cancer cells via a number of pathways, for example, hypoxia, VEGF, oestrogen, and stimulation of the transcription factor NF-*κ*B pathway [65, 71, 72]. As previously mentioned, VEGF is also known to stimulate the expression of the IgSF members ICAM-1, VCAM-1, and PECAM-1 [40, 41, 59], so it is possible there may be crosstalk between these molecules and CXCR4. Moreover, Zabouo et al. [2] reported that siRNA-induced downregulation of MCAM was associated with decreased expression of CXCR4 and decreased invasiveness of breast cancer cells. The expression of MCAM and NCAM has also been shown to activate NF-*κ*B in endothelial and myeloid leukemia cell lines, respectively [25, 27].

In addition to a potential role in the regulation of chemokine receptors such as CXCR4, IgSF members themselves may act as an extracellular attractant. Li and Galileo [44] found that soluble L1CAM (sL1), produced by proteolytic cleavage of membrane-bound L1CAM, acted as a chemoattractant for breast cancer cells in transmigration assays and this effect was neutralized using sL1 blocking antibodies.

Lastly, for a nonpolarized and randomly oriented cell to migrate in response to a chemotactic stimulus such as CXCL12, it must display both front-rear polarization and direction sensing [73]. This is a complex process involving a large number of different molecules [73, 74] several of which have been linked with members of the IgSF. For example, melanoma cells exposed to Wnt5a (a cell polarity-associated signaling molecule) in the presence of a chemokine gradient formed an intracellular structure containing actin, myosin, and MCAM. This structure triggered membrane contractility and influenced the direction of cell movement [39]. MCAM has also been implicated in a reciprocal regulatory loop with AKT/PKB (protein kinase b), a molecule that has been associated with increased survival and directional migration in breast cancer cells [42]. In melanoma cells, phosphatidylinositol 3 kinase (PI3K) was found to upregulate MCAM expression via AKT expression and over-expression of MCAM also activated endogenous AKT [43]. It therefore appears that MCAM contributes to directional cell migration via several pathways.

### 5.3. Matrix Degradation

Although the extracellular matrix (ECM) serves as a niche for tumour cells to survive and proliferate, it is also a barrier to cell migration. Thus, degradation of the ECM is one of the first steps in tumour invasion and metastasis [75]. There are many types of proteases involved in ECM degradation, but the matrix metalloproteinases (MMPs) play a key role in metastasis and are upregulated in almost every type of human cancer [76]. Although more than 20 MMPs have been identified to date, the expression and activity of MMP-2 and MMP-9 are most frequently elevated in cancer and have been correlated with increased metastasis and poor prognosis [75]. MMP expression is regulated by both gene transcription and protein modification and activation.

At the transcriptional level, factors that can increase expression of MMP genes include growth factors, cytokines, hormones and the expression of other tumour promoting molecules such as IgSF members [77]. For example, in melanoma, elevated MMP-2 expression has been associated with increased levels of MCAM and NCAM. Forced expression of MCAM in MCAM-negative melanoma cells led to a significant increase in MMP-2 expression [35], and inhibition of MCAM using blocking antibodies decreased the expression of MMP-2 [36]. The mechanism behind the MCAM-MMP-2 axis was recently described by Zigler et al. [45], who found that MCAM regulated the expression of inhibitor of DNA binding-1 (Id-1), a transcription regulator, and that Id-1 expression controls MMP-2 transcription. In addition, Shi et al. [78] reported that the proinvasive function of NCAM is mediated through stimulation of both cyclic adenosine monophosphate (c-AMP) protein kinase (PKA) and PI3K/AKT pathways, which converge at the transcription factor CREB and increase MMP2 expression. Interestingly, CREB activity also upregulates the expression of MCAM [47], which suggests that MCAM may act as a downstream mediator of NCAM.

MMPs are also extensively regulated posttranslationally, as they are synthesized as preproenzymes and activated by proteolytic cleavage. Activation of most MMPs occurs in the extracellular space by serine proteases (e.g., plasmin and urokinase plasminogen activator) or by cell-surface membrane type (MT) MMPs such as MT1-MMP, a potent activator of pro-MMP2 [77]. It is also known that clustering of cell surface receptors such as *β*1 and *α*v*β*3 integrins activates MMP2 [46]. Interestingly, recent data suggests that cell-cell contacts may influence the activation status of MMPs, with less confluent cells showing decreased MMP activity. Lunter et al. [46] found that cell-cell contacts, ALCAM and cell-matrix interactions were all critical for MMP2 activation, as cells transfected with truncated ALCAM showed less cell-cell adhesion and decreased MMP-2 activity due to reduced transcript levels and decreased processing of MT1-MMP.

## 6. Dissemination

The next step in metastasis is the dissemination of tumour cells via the systemic circulation. Intravasation of tumour cells is not well understood, but it is generally believed that tumour cells can pass easily into the irregular, highly permeable blood vessels formed during tumour angiogenesis [79]. Once inside the vasculature, less than 0.1% of these circulating tumour cells (CTCs) are estimated to remain viable after 24 hours and less than 0.01% survive to generate metastases [80]. This may be due to anoikis, the result of fluid shear forces, or immunological attack [79]. Anoikis is an apoptotic process triggered by the loss of cell-matrix interactions and the ability to overcome this is crucial for CTC survival [81]. The loss of cell-matrix attachment disrupts integrin receptors and results in the deactivation of focal adhesion kinase (FAK) and Src family kinases. This leads to the attenuation of prosurvival pathways, the upregulation of proapoptotic proteins, and the initiation of apoptosis [82].

Resistance to anoikis can be conferred by diverse mechanisms, including constitutive activation of FAK, epidermal-growth-factor-receptor- (EGFR-) mediated Src activation, and any disturbance to the apoptotic pathway. Although there is limited evidence that IgSF members confer resistance to anoikis, it is possible they do—firstly, by their ability to provide antiapoptotic signals (as described above in *Apoptotic Evasion) *and secondly, through activation of FAK. Anfosso et al. [48] found that MCAM recruits the protein tyrosine kinase (PTK) FYN to its cytoplasmic tail, leading to the activation of downstream targets such as FAK. Thus, if tumour cells in the vasculature are present as a group (e.g., via collective migration), it is possible that cell-cell interactions mediated by MCAM may upregulate FAK and protect the cells from anoikis. L1CAM expression in ovarian carcinoma cells has also been linked with sustained phosphorylation of FAK and resistance to apoptosis [49].

## 7. Extravasation

The presence of CTCs within the vasculature is common in patients with advanced primary tumours, but these cells do not cause metastatic disease and subsequently exit the circulation [83]. One theory proposed to explain how tumour cells became lodged in the vasculature is that of mechanical entrapment where large tumour cells become stuck in the small vessels of capillary beds and then extravasate into surrounding tissue. This theory is supported by data showing that tumour cells that form homotypic aggregates are likely to be easily trapped in small capillaries and tend to exhibit higher metastatic potential than cells that do not form multicellular aggregates [84, 85]. MCAM, ALCAM, NCAM, and L1CAM have all been implicated in the formation of large cell aggregates and have been shown to increase the metastatic capability of tumour cells [8, 14, 36–38, 50].

However, it seems clear that mechanical entrapment is not the only factor influencing the site of extravasation. If this was the case, tumour cells or cell aggregates would become trapped in the first capillary bed they encounter after being released into the venous circulation; in most cases this would be the lung [83]. Although the lung is a common site of metastases, CTCs also colonize other organs, suggesting that a significant number of tumour cells escape arrest in the pulmonary microcirculation. A recent report showing that cancer cells are capable of adjusting their shape to pass through narrow vessels supports this conclusion [86]. Furthermore, a number of studies have shown that tumour cells can adhere to the walls of precapillary arterioles, whose diameters far exceed cell size [87, 88]. Taken together, these data suggest that specific adhesion occurs between tumour cells and vascular endothelial cells and that the arrest of tumour cells in the capillary beds of particular organs is likely due to a combination of both mechanical trapping and cancer-cell adhesion to specific molecules on the vasculature [83, 86].

Glinskii et al. [86] propose a multistep model of tumour-endothelial cell adhesion, where carbohydrate-lectin interactions, which tend to be weak and transient, initiate an adhesion cascade that subsequently involves more stable interactions. Specifically, they suggest that the Thomsen-Friedenreich (TF) glycoantigen (a *β*-galactoside) on tumour cells leads to clustering of galectin-3 on the surface of endothelial cells and transient adhesion. The association of endothelial galectin-3 with *α*3*β*1 integrin [89] on the tumor cells then stabilizes this adhesion and may mediate multiple downstream signals that determine the fate of the cell deposit and organ-specific metastasis. This work involved primarily breast and prostate cancer cells in bone vasculature and lung vasculature. As it is known that the endothelia in different organs express different cell-surface receptors [90], it is probable that different glycosylation structures and/or different integrins may mediate tumour-endothelial cell interactions in different capillary beds.

Cell-surface glycosylation is upregulated in many different cancers [91], and a number of glycoproteins have been identified as ligands for galectin-3, including integrin *β*1, lysosome-associated membrane proteins 1 and 2 [92], and the IgSF members carcinoembryonic antigen (CEA) and L1CAM [51, 52]. There is also evidence that MCAM expression facilitates melanoma-endothelial cell adhesion [35, 36] although it is not known if this is mediated via carbohydrate or protein binding. In addition, PECAM-1 is located at the cell junctions on endothelial cells and may also contribute to tumour cell arrest and extravasation. PECAM-1 has been described as engaging in both homophilic and heterophilic adhesive interactions, and it is possible that the interaction of PECAM-1 with heparan sulfate proteoglycans on tumour cells could contribute to extravasation. Carcinoma, melanoma, lymphoma, and leukemia cells have been described as overexpressing heparan sulfates of the glypican family compared to that seen in their normal counterparts [53]. Although the possibility that PECAM-1 bound heparan sulfate was controversial for many years, it is now clear that the hypoxic conditions found in tumours would favour this interaction [54]. Thus, IgSF members may contribute to the arrest of tumour cells via both cell aggregation (leading to mechanical trapping) and specific tumour-endothelial cell adhesion.

Most models of metastasis propose that extravasation occurs soon after cell arrest, by degradation of the endothelial basement membrane and the surrounding ECM [93]. However, Al-Mehdi et al. [87] propose that tumour cells may also proliferate intravascularly to form metastatic foci without the need for extravasation. In time, these metastatic colonies are likely to outgrow the vessels, destroy the vascular walls, and invade the surrounding tissue [83].

## 8. Colonization and Proliferation

It is well known that different cancers show an organ-specific pattern of metastases. This is probably due to, firstly, the lodgment of cells in the vasculature, as a result of both entrapment and specific adhesion and, secondly, the ability of the cancer cells to grow in their new environment. Many of the features that allow tumour cells to proliferate in the primary lesion (e.g., apoptotic evasion and the ability to move through the ECM) will also be essential for growth as a secondary lesion. However, metastasizing cells must also adapt to a new microenvironment that is likely to be very different from that of the primary tumour, and their ability to do this will influence whether or not secondary tumours successfully develop at the site of extravasation. The metastasizing cells will need to respond to growth factors and cytokines in the host tissue, proliferate, recruit the necessary supportive stromal cells, and develop an appropriate blood supply [83]. Indeed, all of the characteristics required to facilitate growth of the primary tumour will also be required for the development of a successful metastatic lesion. It is expected that the contribution of IgSF members to these processes in the metastatic lesion will be as described for proliferation of the primary tumour.

The occurrence of metastases of metastases should not be discounted and for some tumours (e.g., melanoma) they may be expected. For the clinician it is of little consequence whether a metastatic lesion arose from the primary tumour or from another metastatic lesion, as the difficulties of treatment are similar. However, from a drug development perspective if metastases of metastases are a possibility, adhesion molecules like MCAM on melanoma, for example, remain a viable drug target even after the first metastases have been diagnosed.

## 9. Immunological Escape

Over a century ago, Paul Ehrlich hypothesized that cancer would be more common in long-lived organisms if the immune system did not protect against cancer (described in [94]). However, it was not until the 1990s, with improved mouse models of immunodeficiency, that the role of cancer immunosurveillance by the immune system was determined. It became clear that those mice lacking the cells of the adaptive immune system (T and B cells) and natural killer (NK) cells were more susceptible to tumour formation and dissemination [94, 95]. As our knowledge has increased, it has become apparent that, although cancer immunology is very complex, NK cells and T and B lymphocytes are able to recognize tumour cells as abnormal and target them for destruction (the elimination phase) [94]. However, it appears that rare cancer cell variants survive elimination by the immune system and that tumour cell clusters increase the probability of cells being protected from immunological attack. These tumour cell clusters are held in check by the immune system but not all the cells are destroyed (equilibrium or dormant phase). This dormant phase can last for years, until the tumour cells acquire the ability to escape immune recognition or there is a change in the immune system of the host [94].

While there is no evidence that IgSF members directly lead to immunoescape of tumour cells, molecules such as MCAM, ALCAM, and NCAM mediate cell-cell cohesion, enabling the formation of cell aggregates [36, 37, 50]. It is believed that the formation of tumour aggregates ensures the survival of the inner cells, particularly during migration and dissemination, as the outer cells protect them from the immune-mediated cell death [16, 18].

## 10. Conclusion

The metastatic cascade is very complex and most research in this area has focused on the role of integrins and cadherins in cell migration and invasion, using carcinoma as a model system. In writing this paper, our goal was to examine the potential role of a selection of IgSF members in the metastatic pathway in different types of cancer, including carcinoma, melanoma, and sarcoma. Although most of these molecules have been described as tumour biomarkers, the extent and nature of their contribution to the metastatic pathway has not been clear. We have examined aspects of each step in the pathway and have suggested ways in which one or more of the six IgSF members could contribute. Much of this is conjecture based on what is known about the behaviour of these proteins in nontumour systems. However, as tumours commonly use existing molecular interactions in inappropriate or aberrant ways, we feel our conclusions indicate some interesting possibilities for further research. Performing these studies, however, will not be easy because of the difficulties of accurately dissecting a system as complex as the metastatic cascade *in vivo *and the limitations of the *in vitro* assays used to support *in vivo* conclusions. It is for these reasons that much remains to be understood, particularly about the role of IgSF members in the metastatic cascade. Yet the need to understand metastasis is high because most patients that succumb to cancer succumb to metastasis or the complications of its treatment. 

## Figures and Tables

**Table 1:** The role of IgSF members in the metastatic cascade.

Stages in metastasis	Involvement of IgSF members	
	Known role	Potential role
(1) Cell proliferation in primary tumour		
(i) Apoptotic	NCAM [24]	MCAM [27, 28]
evasion	ALCAM [29]	
(ii) Angiogenesis	PECAM-1 [30, 31]	VCAM [32]
	ICAM-1 [33, 34]	

(2) Local cell invasion		
	MCAM [8, 35, 36]	
(i) Cell-cell	ALCAM [37]	
interactions	L1CAM [14, 38]	
		ICAM-1 [40]
(ii) Directional cell		VCAM-1 [40]
migration and cell	MCAM [39]	PECAM-1 [41]
polarity		NCAM [27]
		MCAM [25, 42, 43]
		L1-CAM [44]
(iii) Matrix	MCAM [35, 36, 45]	ALCAM [46]
degradation	NCAM [47]	

(3) Intravasation and dissemination		MCAM [48]
		ALCAM [49]

(4) Extravasation		MCAM [8, 35, 36]
		ALCAM [14, 37]
		NCAM [50]
		L1CAM [38, 51, 52]
		PECAM-1 [53, 54]

(5) Colonization and proliferation	As for (1) and (2)	As for (1) and (2)

(6) Immunological escape		MCAM [8, 36]
		ALCAM [14, 37]
		NCAM [50]
